# Supplementary figures and images for: Rad51 filaments assembled in the absence of the complex formed by the Rad51 paralogs Rad55 and Rad57 are outcompeted by translesion DNA polymerases on UV-induced ssDNA gaps
Source: PLoS Genet. 2023 Feb 7;19(2):e1010639. doi: 10.1371/journal.pgen.1010639 (PMC9937489; doi:10.1371/journal.pgen.1010639)

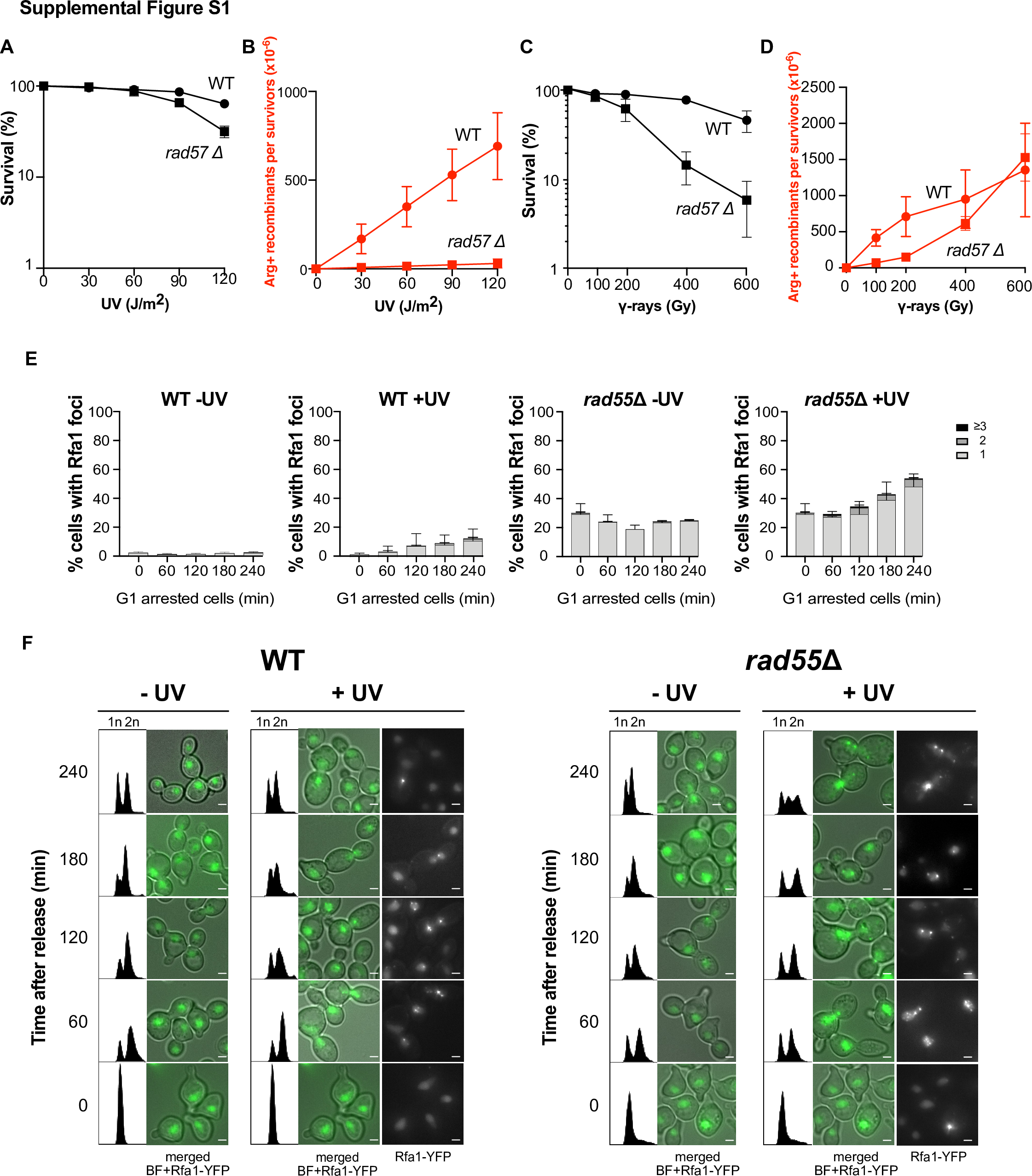

Supplement: S1 Fig — (A) Survival, and (B) [Arg+] recombinant frequencies in WT and rad57Δ diploid cells following UV radiation. (C) Survival and (D) [Arg+] recombinant frequencies in WT and rad57Δ diploid cells after γ irradiation. (E) Quantification of Rfa1-YFP foci in WT and rad55Δ cells not irradiated or after UV exposure in G1-arrested cells. Error bars indicate SDs from three independent experiments. (F) Representative images of Rfa1-YFP WT and rad55Δ cells released from G1 arrest and UV-irradiated or not. Bright-field (BF) images are merged with YFP images. Scale bars are 2 μm. FACS profiles for each corresponding time point are shown. (TIF) [file pgen.1010639.s001.tif]

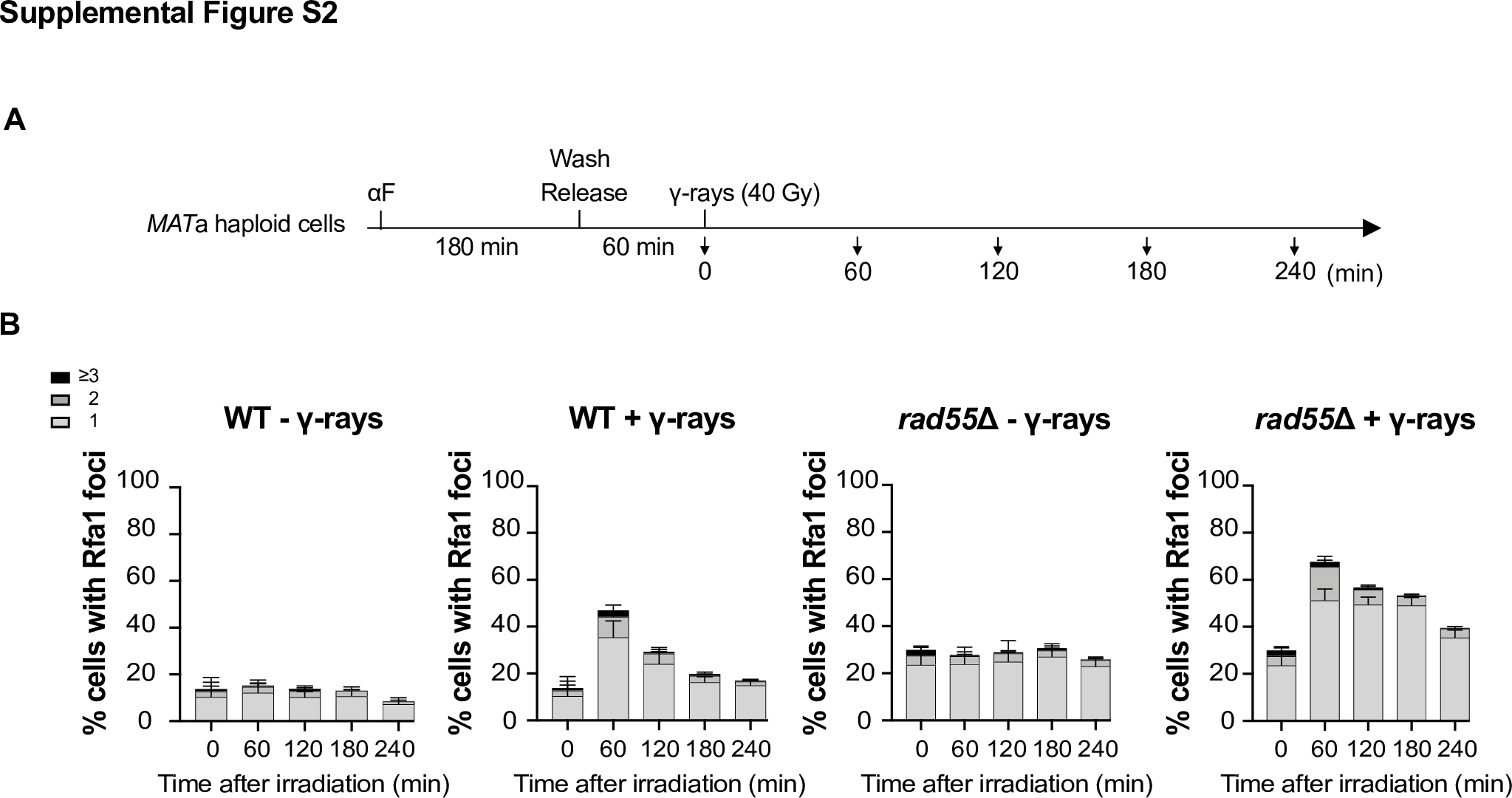

Supplement: S2 Fig — (A) Experimental scheme: Cells arrested in G1 phase with alpha-factor (αF) were release into the cell cycle. After one hour from the release, cells were exposed to γ-rays. Samples were collected every hour for four hours. (B) Quantification of Rfa1-YFP foci in WT and rad55Δ cells not irradiated or after γ-rays irradiation. Error bars indicate SDs from three independent experiments. (TIF) [file pgen.1010639.s002.tif]

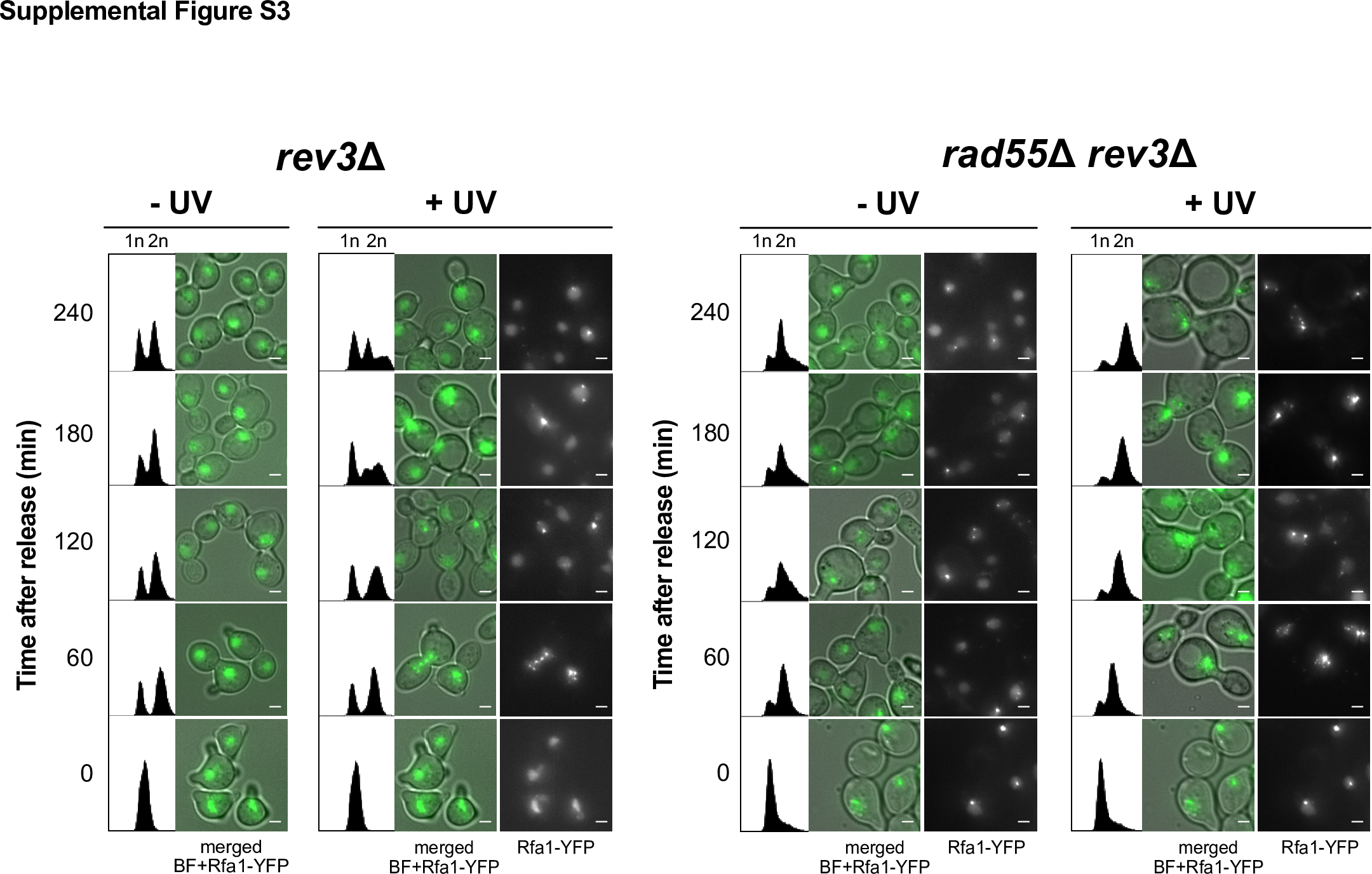

Supplement: S3 Fig — Representative images of Rfa1-YFP rev3Δ and rad55Δ rev3Δ cells after release from G1 arrest and UV irradiated or not. Bright-field (BF) images are merged with YFP images. Scale bars are 2 μm. FACS profiles for each corresponding time point are shown. (TIF) [file pgen.1010639.s003.tif]

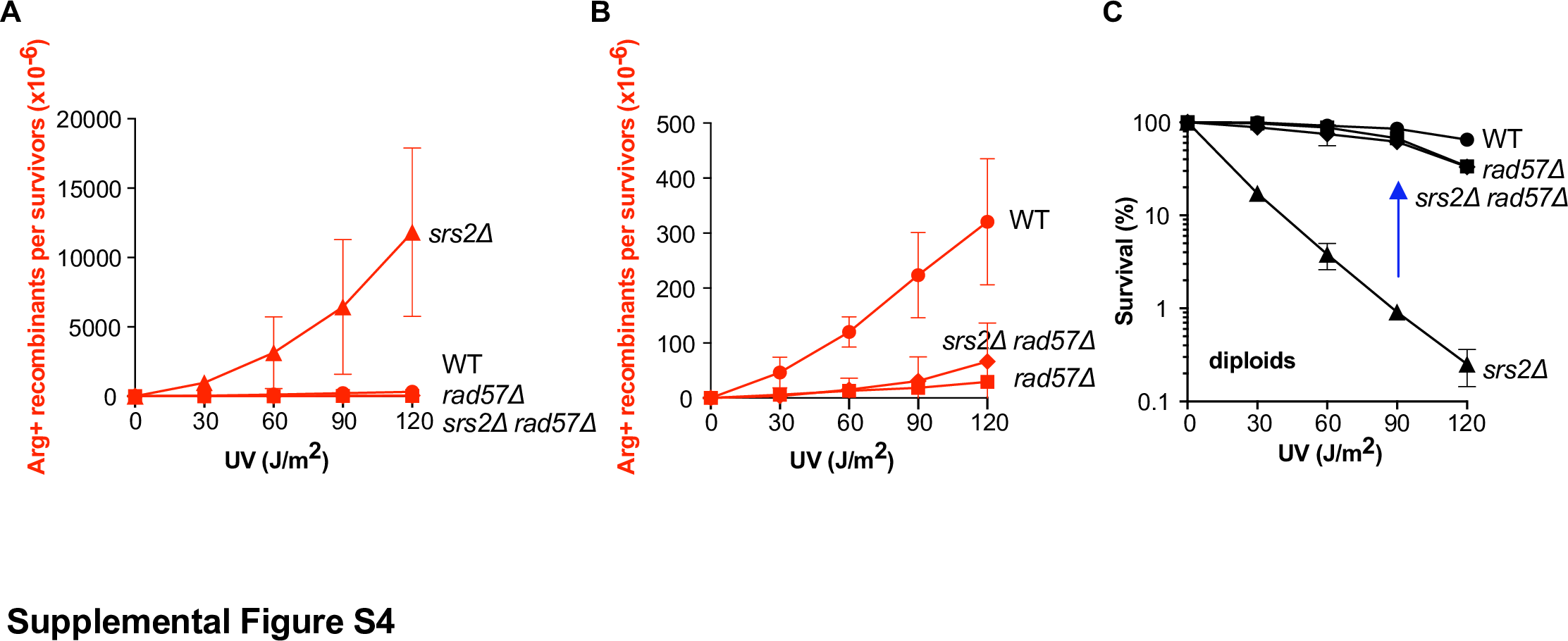

Supplement: S4 Fig — (A) UV-induced [Arg+] recombinant frequencies in rad57Δ srs2Δ diploid cells. (B) Close-up view of (A) to exclude the hyper-recombinogenic phenotype of srs2Δ. (C) Survival curves of diploid cells following UV radiation. The acute sensitivity to UV radiation of the diploid srs2Δ strain is suppressed by rad57Δ (blue arrow). (TIF) [file pgen.1010639.s004.tif]

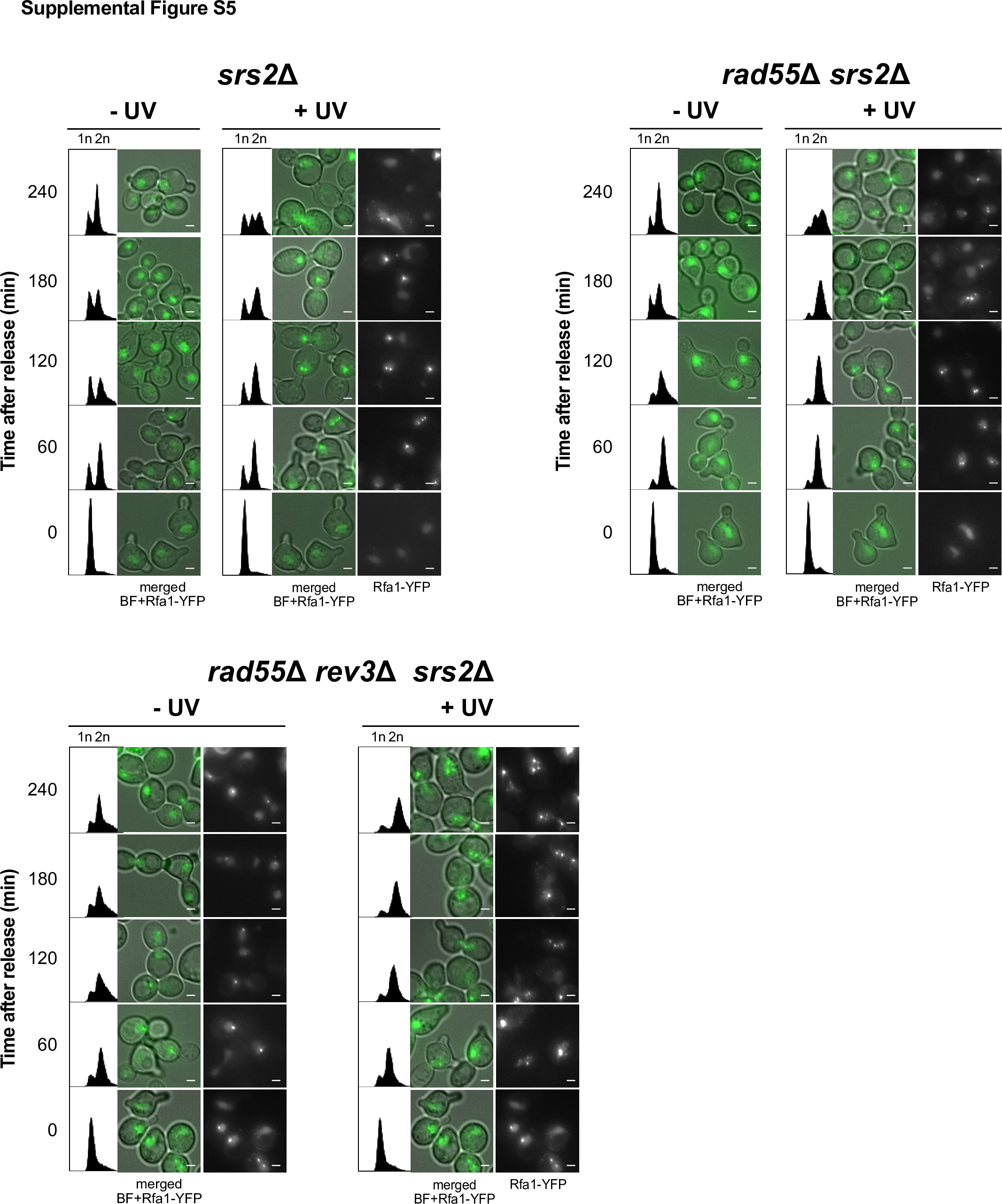

Supplement: S5 Fig — Representative images of Rfa1-YFP srs2Δ, rev3Δ srs2Δ and rad55Δ rev3Δ srs2Δ cells after release from G1 arrest and UV irradiated or not. Bright-field (BF) images are merged with YFP images. Scale bars are 2 μm. FACS profiles for each corresponding time point are shown. (TIF) [file pgen.1010639.s005.tif]
