# Supplementary material for: Rad51 filaments assembled in the absence of the complex formed by the Rad51 paralogs Rad55 and Rad57 are outcompeted by translesion DNA polymerases on UV-induced ssDNA gaps
Source: PLoS Genet. 2023 Feb 7;19(2):e1010639. doi: 10.1371/journal.pgen.1010639 (PMC9937489; doi:10.1371/journal.pgen.1010639)
Supplement: S1 Table — (PDF) [file pgen.1010639.s006.pdf]

Table S1

Strain name	Ploidy	Relevant genotype	Figure	Background	Source
FF18733	1n	*MAT***a** *leu2-3, 112 trp1-289 ura3-52 lys1-1 his7-2*		FF	F. Fabre
W303	1n	*MAT***a** *ADE2 leu2-3,112 his3-11,15 trp1-1, ura3-1*		W303	R. Rothstein
Diploids	2n	**a**/α *arg4-RV / arg4-Bg*		FF	
DLM218	2n	**a**/α WT	Fig. 1B-C-D-E	FF	
DLM158	2n	**a**/α *rad55::URA3*	Fig. 1B-C-D-E	FF	
DLM143	2n	**a**/α WT	Fig. 1F	FF	
DLM198	2n	**a**/α *rad55::URA3*	Fig. 1F	FF	
yKD577	1n	**a** *bar1::LEU2 RFA1-YFP*	Fig. 1G-H; S1 Fig. F; S2 Fig.	W303	R. Rothstein
L433.1D	1n	**a** *rad55::NAT bar1::LEU2 RFA1-YFP*	Fig. 1G-H; S1 Fig. F; S2 Fig.	W303	
L317.4A	1n	**a** WT	Fig. 2A	FF	
L323.11A	1n	**a** *rad55::URA3*	Fig. 2A	FF	
L364.11B	1n	α *rev3::URA3*	Fig. 2A	FF	
L364.2A	1n	α *rad55::URA3 rev3::URA3*	Fig. 2A	FF	
DLM143	2n	**a**/α WT	Fig. 2B	FF	
DLM158	2n	**a**/α *rad55::URA3*	Fig. 2B	FF	
DLM199	2n	**a**/α *rev3::URA3*	Fig. 2B	FF	
DLM200	2n	**a**/α *rad55::URA3 rev3::URA3*	Fig. 2B	FF	
FF18733	1n	**a** WT	Fig. 2C	FF	
L376.2B	1n	**a** *rad55::URA3*	Fig. 2C	FF	
L430.15D	1n	**a** *rev3::URA3 bar1::LEU2 RFA1-YFP*	Fig. 2D; S3 Fig.	W303	
L433.2C	1n	**a** *rad55::NAT rev3::URA3 bar1::LEU2 RFA1-YFP*	Fig. 2D; S3 Fig.	W303	
DLM218	2n	**a**/α WT	Fig. 3A	FF	
DLM199	2n	**a**/α *rev3::URA3*	Fig. 3A	FF	
DLM214	2n	**a**/α WT	Fig. 3B-C	FF	
DLM212	2n	**a**/α *rad30::KanMX*	Fig. 3B-C	FF	
DLM158	2n	**a**/α *rad55::URA3*	Fig. 4A-E-F	FF	
DLM199	2n	**a**/α *rev3::URA3*	Fig. 4A	FF	
DLM200	2n	**a**/α *rad55::URA3 rev3::URA3*	Fig. 4A	FF	
DLM214	2n	**a**/α WT	Fig. 4B-C-D	FF	
DLM213	2n	**a**/α *rad55::URA3*	Fig. 4B-C-D	FF	
DLM212	2n	**a**/α *rad30::KanMX*	Fig. 4B-C-D	FF	
DLM211	2n	**a**/α *rad55::URA3 rad30::KanMX*	Fig. 4B-C-D	FF	
DLM218	2n	**a**/α WT	Fig. 4E-F	FF	
DLM203	2n	**a**/α *mms2::NAT*	Fig. 4E-F	FF	
DLM204	2n	**a**/α *rad55::URA3 mms2::NAT*	Fig. 4E-F	FF	
DLM218	2n	**a**/α WT	Fig. 5A-B-D	FF	
DLM158	2n	**a**/α *rad55::URA3*	Fig. 5A-B-D	FF	
DLM145	2n	**a**/α *srs2::LEU2*	Fig. 5A-D	FF	
DLM160	2n	**a**/α *rad55::URA3 srs2::LEU2*	Fig. 5A-B-D	FF	
DLM143	2n	**a**/α WT	Fig. 5C	FF	
DLM199	2n	**a**/α *rev3::URA3*	Fig. 5C	FF	
DLM200	2n	**a**/α *rad55::URA3 rev3::URA3*	Fig. 5C	FF	
DLM202	2n	**a**/α *rad55::URA3 rev3::URA3 srs2::LEU2*	Fig. 5C	FF	
L436.1A	1n	**a** *srs2::HIS3 bar1::LEU2 RFA1-YFP*	Fig. 5E; S5 Fig.	W303	
L436.7A	1n	**a** *rad55::NAT srs2::HIS3 bar1::LEU2 RFA1-YFP*	Fig. 5E; S5 Fig.	W303	
TLM305	1n	**a** *rad55::NAT rev3::URA3 srs2::HIS3 bar1::LEU2 RFA1-YFP*	Fig. 5E; S5 Fig.	W303	
JKM146	1n	*hml::ADE1 MATalpha hmr::ADE1 arg5,6::MATa-inc::HPH1 ade3::GAL::HO*			J. Haber
EC2604	1n	WT	Fig. 6B	JKM146	
EMY149	1n	*rad55::URA3*	Fig. 6B	JKM146	
TLM261	1n	*rev3::KanMX*	Fig. 6B	JKM146	
TLM277	1n	*rad30::URA3*	Fig. 6B	JKM146	
TLM281	1n	*rev3::KanMX rad30::URA3*	Fig. 6B	JKM146	
TLM262	1n	*rad55::NAT rev3::KanMX*	Fig. 6B	JKM146	
TLM278	1n	*rad55::NAT rad30::URA3*	Fig. 6B	JKM146	
TLM288	1n	*rad55::NAT rev3::KanMX rad30::URA3*	Fig. 6B	JKM146	
DLM218	2n	**a**/α WT	S1 Fig.; S4 Fig.	FF	
DLM219	2n	**a**/α *rad57::NAT*	S1 Fig.; S4 Fig.	FF	
DLM145	2n	**a**/α *srs2::LEU2*	S4 Fig.	FF	
DLM221	2n	**a**/α *rad57::NAT srs2::LEU2*	S4 Fig.	FF	
