# Supplementary material for: Rad51 filaments assembled in the absence of the complex formed by the Rad51 paralogs Rad55 and Rad57 are outcompeted by translesion DNA polymerases on UV-induced ssDNA gaps
Source: PLoS Genet. 2023 Feb 7;19(2):e1010639. doi: 10.1371/journal.pgen.1010639 (PMC9937489; doi:10.1371/journal.pgen.1010639)
Supplement: Attachment — Submitted filename: Review Maloisel et al PLOS GENET 2022.docx [file pgen.1010639.s007.docx]

**COMMENTS**

The authors show nicely and convincingly in yeast S cerevisiae that the complex formed by the Rad55/Rad57 complex is essential for Rad51 filament formation at UV-induced ssDNA gaps, and only marginal at DSB sites, with a strong decrease of HR frequencies in the rad55Δ and rad57Δ mutants specifically after UV radiation. They also show that depletion of both Rad55 and TLS polymerase Polζ or Polη increases synergistically UV sensitivity and not sensitivity to the induction of site-directed DSB. They also reported increased UV-induced mutagenesis in the rad55Δ mutant due to TLS polymerases and that in absence of TLS polymerase, UV-induced HR is increased. The interplay between the Rad55/Rad57 complex and the Srs2 anti recombinase is less obvious and more confused, although the results suggest that the Rad55-Rad57 complex protects Rad51 filaments against Srs2 at UV-induced ssDNA gaps

The data are novel interesting, the experimental methods are rigorous, and the manuscript is generally well written. This study warrants publication in PLOS Genetics. There are just some issues, listed below, the authors should address before this work is published

1) The authors claim that UV-induced HR is triggered by ssDNA gaps by referring to previous published works. Would it be possible for the authors to demonstrate in an easily way that generation of ssDNA gaps by UV is true in their own cellular models mutants? What would be the mechanism involved in the formation of the gaps behind moving replication forks? Is Primpol or equivalent expressed in S cerevisiae?
2) The control treatment compared to UV is the treatment with IR which, as claimed by the authors, generates DSBs and ssDNA gaps. I suppose that single strand breaks and the multiple oxidative DNA damage can also be generated by such an irradiation, and may interfere with HR and TLS processes. Could the authors comment on this issue?
3) The results here suggest that in the absence of Rad55-Rad57, unstable Rad51 filaments cannot prevent the loading of the TLS polymerases, but what would be the mechanism of loading in the presence of RAD51 for Polζ and Polη ?. In human cells, it has been evidenced the ability of Polθ in removing Rad51 and RPA, but there is no evidence for other TLS polymerases to perform this task.
4) Did the authors test other TLS DNA polymerases ? Why only Polζ and Polη?
5) In human cells; mutations in [BRCA1](https://www.sciencedirect.com/topics/biochemistry-genetics-and-molecular-biology/brca1) or [BRCA2](https://www.sciencedirect.com/topics/biochemistry-genetics-and-molecular-biology/brca2) (BRCA) is synthetic lethal with poly(ADP-ribose) polymerase inhibitors (PARPi) and such synthetic lethality is thought to derive from DSB repair BRCA function. It was recently reported by the Sharon Cantor’s lab (Cancer Res 2021, Mol cell 2021) instead that toxicity derives from replication gaps which characterize BRCA-deficient cells, are diminished upon resistance and restored upon resensitization. Such gaps are determinant of PARPi toxicity changing the paradigm for synthetic lethal interactions. These observations could be related to the present work and might be discussed.
