# Supplementary material for: Rad51 filaments assembled in the absence of the complex formed by the Rad51 paralogs Rad55 and Rad57 are outcompeted by translesion DNA polymerases on UV-induced ssDNA gaps
Source: PLoS Genet. 2023 Feb 7;19(2):e1010639. doi: 10.1371/journal.pgen.1010639 (PMC9937489; doi:10.1371/journal.pgen.1010639)
Supplement: Attachment — Submitted filename: Response to reviewers.docx [file pgen.1010639.s008.docx]

Fontenay-aux-Roses, January 12^th^, 2023

Dear Lorraine, Dear Gregory,

Thank you very much for considering a revised version of our manuscript entitled “Rad51 filaments assembled in the absence of the complex formed by the Rad51 paralogs Rad55 and Rad57 are outcompeted by translesion DNA polymerases on UV-induced ssDNA gaps” for publication in PLOS Genetics. We have taken your advice and comments into account and have made an extensive revision of the manuscript. We would like to thank you and the Reviewers for your efforts and careful consideration. You have given us thoughtful and very helpful suggestions on how to improve the manuscript. We hope that our clarifications and our additional experiments address all of your concerns as well as those raised by the reviewers. We completed a large number of microscopy experiments working in collaboration with Karine Dubrana and Jamie Phipps, as well as Chromatin Endogenous Cleavage (ChEC) experiments and CHEF gels, completed in collaboration with Stephane Marcand, Stefano Mattarocci and Alice Deshayes. Even if only the microscopy experiments were successful, they all realized a large quantity of works and we consider that they should be all added as authors in this new version. We hope that will suit you. We had to ask for extra time to complete these experiments and we apologize for the delay. We are providing below, a separate list of point-by-point responses to the reviewers.

Kind regards,

Reviewer #1: In this pithy manuscript by Maloisel et al, the authors examined the role of Rad55 in UV-induced recombination. They showed that despite not being UV sensitive, rad55∆ cells are unable to perform allelic recombination upon UV damage. This is due to TLS bypassing these lesions in an error-prone manner. The authors argue that genetic relationship between RAD55 with REV3 and RAD30 is related to gap repair and not DSB repair. The novelty of this study is the analysis of UV-induced damage and the finding that HR is needed to bypass UV-induced gaps and not direct DSBs. A few experiments detailed below would help provide more direct evidence and mechanistic understanding for the genetic observations presented.

1. The authors propose that Rad55-Rad57 stabilize Rad51 filaments independently of the protection against Srs2. Experimental evidence (such as ChIP or fluorescent microscopy) to support this model is warranted.

Detection of Rad51 around origins of replication in a population of cells released from a S-phase arrest and exposed or not to UV-irradiation would be ideal to answer this point. Therefore, we measured the recruitment of Rad51 by ChIP assay at the sites of an early firing origins (ARS306) one hour after release from the alpha-factor arrest, cells being irradiated at 60 J/m^2^ at the time of the release. Polyclonal Rad51 antibodies from Abcam was used. Unfortunately, we observed no enrichment of Rad51 at four PCR amplified sites located at 0, 1, 4 and 6 kb from ARS306 in irradiated or unirradiated cells.

We also used chromatin fractionation assays to catch an enrichment of Rad51 to the chromatin after UV-irradiation. Cells were arrested with alpha-factor and released after UV-irradiation at 60J/m^2^. Cells were collected at 1- and 2-hour time points after release and cell extracts were performed by detergent treatment of spheroplasts, accordingly to the protocol described in (Cabello-Lobato, MJ et al, 2021). Cell extracts were centrifuged for 15 minutes at 13 000 g to generate chromatin fractions. The amount of Rad51 present in the chromatin fraction was not increased after UV-irradiation making this technique impossible to use as a read-out for the control of Rad51 filament formation.

Consequently, we turned to microscopy to monitor the repair events taking place after UV-irradiation. We decided not to directly observe Rad51 foci formation, as a functional fluorescent version of the Rad51 protein was not yet available. Therefore, we decided to monitor repair events through the formation of UV-induced RPA foci, representative of ssDNA accumulation after UV-irradiation (Wong et al., Moll. Cell., 2019). Rfa1-YFP fusion protein was used to test first if accumulation of RPA foci would be observed upon UV-irradiation in *rad55*∆ mutant cells. As now shown in Fig. 1G&H, 60% of cells exposed at 60 J/M^2^ of UV after release from an alpha factor G1 arrest display the formation of RPA foci one hour after irradiation, the number of foci decreasing with time to reach 20% of cells four hours after exposure. On the contrary, 80% of *rad55*∆ cells show RPA foci one hour after exposure and 60% still shows RPA foci four hours after treatment. This delay in RPA foci resolution indicates that single-strand gaps accumulate in *rad55*∆ cells, confirming our conclusion that Rad55 is important to promote UV-induced HR (Fig. 1C).

The same experiment shows that *rad55*∆ *srs2*∆ cells behave like *rad55*∆ cells, showing a slower disappearance of RPA foci. We also observed that the deletion of *SRS2* in *rad55*∆ r*ev3*∆ cells does not reduce the number of RPA foci observed, even if cells survival was significantly increased (Fig. 5C). This confirms that Rad55 requirement in UV-induced HR is largely Srs2-independent as shown on (Fig 5).

2. As suggested in the discussion, it would greatly improve the mechanistic insight of the study to test the model that Rad55-Rad57 directly prevents TLS polymerase recruitment (such as ChIP or fluorescent microscopy).

In the discussion section, we effectively proposed L379: “Thus, in our model, Rad51 filaments formed with the Rad55-Rad57 complex preserve the genome stability through ssDNA gap repair by HR, but also through direct competition with TLS on common ssDNA gap substrates.” In fact, we did not mean that there was a physical interaction between Rad55-Rad57 and the TLS. As the previous sentence says: “Third, the Rad55-Rad57 complex inhibits TLS polymerases by preventing their loading at the primer/template junction within ssDNA gaps, probably through the stabilization of Rad51 filaments”, we are more willing to think that stabilization of Rad51 filaments formed at the site of the lesions impedes TLS recruitment. It would be very interesting to see evidence of the competition between TLS and Rad55-Rad57 with molecular evidence. However, TLS recruitment to the site of a DNA lesion might be transient and therefore difficult to detect. This is unfortunately what we found using several assays.

First, we tried to measure TLS recruitment by ChIP experiment in UV-irradiated cells carrying a TLS *REV1-9MYC* allele with the same protocol used for Rad51 ChIP. We found that the tagged protein is functional, but we could not find any enrichment around the site of the early origin ARS306. We used the same construct to measure if the amount of Rev1-9MYC would increase upon UV-exposure in a precipitated chromatin fraction. Rev1-9MYC is easily detected in the chromatin fraction by western blot, but we could not observe any enrichment after UV-exposure, making it impossible to measure the effect of *rad55*∆ mutant. We also tried to measure the recruitment of Rev1 with the Chromatin Endogenous Cleavage technique (ChEC, Schmid et al, Moll; Cell. 2004). We constructed fusion of the Rev1 protein with the micrococcal nuclease (MNase), that we found to be functional. Again, we could not see any increase in DNA digestion after UV-exposure in the same condition we used for ChIP: α-factor arrest, UV-irradiation, release in cell cycle and cell extraction at 1 and 2h. We then again turned to microscopy, trying to detect Rev1-GFP or Rev1-GFP-GFP foci formation upon UV-exposure, but did not observe any focus.

However, measurement of the number of RPA foci forming after UV-exposure in *rad55*∆ *rev3*∆ double mutants clearly show an additive effect (Fig. 2D). Nearly all cells display at least one RPA foci, with 50 % of the population showing at least 3 RPA foci. More than 80% of the cells still have RPA foci four hours after UV-exposure. These results clearly show that TLS can compensate for HR, and can compete for the same substrate.

3. The model figure would be clearer if the consequences of knocking out the individual pathways or both pathways were also indicated. The dotted arrows made it seem like the pathways were still active. Perhaps “x”s could be placed on top of the arrows to show that a particular pathway is blocked.

Fig. 7 shows the consequences of knocking out *rad55*∆, *rev3*∆, *rad30*∆, *rad55*∆ *rev3*∆ and *rad55*∆ *rad30*∆. Actually, we think that the pathways are still active. For example, HR is still partly active in *rad55*∆ *rev3*∆ double mutants. Same thing in *rev3*∆ or *rad30*∆, where Rad30 and Rev3 are still active respectively. For better clarity, we suppressed the red color of arrows as it was misleading.

Reviewer #2: The manuscript entitled “Rad51 filaments assembled in the absence of the complex formed by the Rad51 paralogs Rad55 and Rad57 are outcompeted by translesion DNA polymerases on UV- induced ssDNA gaps” examined the role of Rad55/Rad57 in UV-induced HR and cellular tolerance to UV. While previous studies examined DSB repair of the rad55 and rad57 mutants, this study uncovered the crucial role of Rad55/Rad57 in gap repair. This is an important conclusion, and this manuscript should be publishable in PLoS Genet if appropriate revision is done.

Specific comments:
1. The two Tad51 paralogs facilitate the formation of Rad51 filaments. However, this study did not analyze the formation of Rad51 filaments following UV irradiation. The authors need to show the formation of Rad51 filaments in the rad55 or rad57 mutant comparing X-ray-induced DSBs and UV-induced gaps.

Unfortunately, as described in the response to Reviewer 1, we have not been able to observe Rad51 recruitment after UV-exposure by ChIP or chromatin fractionation. Because a functional Rad51 tagged protein is not yet available, it is not possible to observed Rad51 recruitment to the chromatin by foci formation or even ChEC analysis. However, as explained to Reviewer 1, we have been able to measure the impact of the *rad55*∆ mutation on post-replicative DNA damage bypass by measuring the formation of RPA foci and this is an interesting read-out to compare the role of Rad55 after UV- or γ-exposure. As underlined by Reviewer 3 (point 2), γ-rays does not only generate DSBs but also ssDNA gaps, through the multiple oxidative DNA damage induced. Therefore, even if UV induces very few DSB at the dose employed (60J/m^2^), γ-rays induces both DSB and ssDNA gaps. We found it interesting to compare the behavior of RPA foci after cells-exposure to γ-rays in comparison with UV. In WT cells, we found the same proportion of cells displaying RPA foci after γ-rays exposure, these foci being resolved with the same kinetics than after UV-exposure. However, as already described in (Wong et al., Moll. Cell., 2019), 70% of the γ-rays irradiated cells displayed only 1 focus against 50% of UV-induced cells. This difference is even more pronounced in *rad55*∆ mutant, where 70% of γ-rays irradiated cells show only one focus while only 40% of UV-irradiated cells shows a single focus. These differences could be attributed to the formation of a DSB repair center after γ-rays exposure while single strand gaps are not gathered after UV-irradiation. This result agrees with our conclusion that Rad51 filament are assembled mainly on ssDNA gaps after UV exposure, while events could be gathered in repair centers after γ-rays irradiation. These observations have now been added in the first part of the results section.

2. The title seems to be misleading to general readers. The rad55 and rad57 mutants display only modest UV sensitivity in the presence of functional translesion synthesis (TLS) DNA polymerases while the rad30 and rev3 mutants are hypersensitive to UV (Fig. 2A and B and Fig.3C). A straightforward interpretation is “Rad51 filaments assembled in the PRESENCE of the complex formed by the Rad51 paralogs Rad55 and Rad57 are outcompeted by translesion DNA polymerases on UV- induced ssDNA gaps”. The reviewer believes that the authors’ finding is very important even when the two Tad51 paralogs acts as a backup for the TLS-dependent rapid release from replication blockage at UV-damage. Please consider changing the title.

The fact that *rad55*∆ or *rad57*∆ mutants display only modest UV-sensitivity while the double mutant *rad55*∆ *rev3*∆ is extremely sensitive indicates that TLS can substitute for HR in the absence of Rad55-Rad57. Actually, if Rad51 filaments assembled in the presence of Rad55-Rad57 was outcompeted by TLS, an increase of TLS-dependent mutagenesis would not be observed in *rad55*∆ cells compared to WT.

3. This manuscript is further improved if the authors show the fiber analysis, IdU labeling, UV irradiation, and CldU labeling, comparing wild type, rad55 mutant, rev3 mutant, and rad55/rev3 double mutant, if possible.

We think that our analysis of RPA foci in these mutants provides an appropriate alternative to fiber analysis as we found that RPA foci formation after UV-exposure is increased in *rad55*∆ and *rev3*∆ mutants, with an additive effect in the *rad55*∆ *rev3*∆ double mutant.

4. Please show the survival data of rad55/srs2 double mutant. Is this mutant lethal?

The survival curve for *rad55*∆ *srs2*∆ was already shown on Fig.5D. The raw data were provided in TableS7 (Source data for Fig.5). As shown on this figure, *rad55*∆ mutation suppresses *srs2*∆ cells UV-sensitivity, a result previously observed in cells exposed to γ-rays, as it was explained in the introduction section L112.

5. Page 6 line 131: “Rad55-Rad57 complex is essential for UV-induced HR, but only accessory for IR-induced HR”. Many readers know little about yeast genetic studies, and the authors need to show the data of rad51 mutant in Fig. 1D and Fig. 2 and also the data of rad51/rev3 double mutant in Fig. 2.

We thought that adding these previously published results to the curves in Fig1 and 2 would make them heavier and we find it problematic to present data that were already published. However, we agree that comparing the results we obtained with *rad55*∆ mutants with those previously published with *rad51*∆ would reinforce the validity of our conclusions. We have already stated in the results section that the survival of *rad51*∆ and *rad52*∆ cells was only slightly affected after UV-exposure (L155). In the results section, we compared our *rad55*∆ results with those previously obtained with *rad51*∆ on line 167 and *rad55*∆ *rev3*∆ with *rad51*∆ *rev3*∆ on line 239. HR, as well as mutagenesis, cannot be accurately measured in *rad55*∆ *rev3*∆ cells because the very low survival observed from the lowest dose applied induces a large variability in the number of survivors counted and consequently in HR or mutagenesis frequencies measured in independent experiments.

6. Page 14 line 329: “UV-induced lesions can be channeled from HR to TLS”. It is formally possible that a small portion of the UV-induced gaps are converted to DSBs that trigger a substantial fraction of the heteroallelic HR events (Fig. 1C). Moreover, unrepaired DSBs, but not remaining gaps, lead to cell death (Fig. 1B and D). Fig. 2C argues against this idea by showing that loss of Rad55 increased TLS-mediated mutagenesis and that Rad55 and TLS share the same substrate, single-strand gaps. Fig. 2C appears to be the only data that suggests “channeled from HR to TLS” when functional Rad55 is absent. To show Rad55 and TLS share the same substrate, single-strand gaps, the authors need to add the data of rev3 single mutant and rad55/rev3 double mutants to Fig. 2C.

Again, we are not comfortable with adding previously published results to our Figures but for the sake of clarity, we add in the results section on line 232 that UV-induced mutagenesis was highly dependent on Rev3 activity.

Reviewer #3: My review is uploaded as an attachment

The authors show nicely and convincingly in yeast S cerevisiae that the complex formed by the Rad55/Rad57 complex is essential for Rad51 filament formation at UV-induced ssDNA gaps, and only marginal at DSB sites, with a strong decrease of HR frequencies in the rad55Δ and rad57Δ mutants specifically after UV radiation. They also show that depletion of both Rad55 and TLS polymerase Polζ or Polη increases synergistically UV sensitivity and not sensitivity to the induction of site-directed DSB. They also reported increased UV-induced mutagenesis in the rad55Δ mutant due to TLS polymerases and that in absence of TLS polymerase, UV-induced HR is increased. The interplay between the Rad55/Rad57 complex and the Srs2 anti recombinase is less obvious and more confused, although the results suggest that the Rad55-Rad57 complex protects Rad51 filaments against Srs2 at UV-induced ssDNA gaps

The data are novel interesting, the experimental methods are rigorous, and the manuscript is generally well written. This study warrants publication in PLOS Genetics. There are just some issues, listed below, the authors should address before this work is published

1) The authors claim that UV-induced HR is triggered by ssDNA gaps by referring to previous published works. Would it be possible for the authors to demonstrate in an easily way that generation of ssDNA gaps by UV is true in their own cellular models mutants? What would be the mechanism involved in the formation of the gaps behind moving replication forks? Is Primpol or equivalent expressed in S cerevisiae?

We initially cited the work from (Lopes et al., Moll. Cell., 2006) in the introduction section (line 133) which already showed the generation of ssDNA gaps by UV in *S. cerevisiae*. Recently, the work of (Wong et al., Moll. Cell., 2019), using RPA as a read-out of the formation of UV-induced ssDNA gaps reinforced the idea that UV generated mostly ssDNA gaps. The large number of *rad18*∆ cells accumulating RPA foci upon exposure to UV is very illustrative, DSBs usually being assembled in a repair center. We added this reference and explained which mechanisms could be involved in the formation of gaps into the introduction section for clarity. In the new version of the manuscript, we used the same read-out to explore the competition between TLS and HR. No primpol activity has been described in yeast yet.

2) The control treatment compared to UV is the treatment with IR which, as claimed by the authors, generates DSBs and ssDNA gaps. I suppose that single strand breaks and the multiple oxidative DNA damage can also be generated by such an irradiation, and may interfere with HR and TLS processes. Could the authors comment on this issue?

This is totally true and the reason why it is not always easy to understand phenotypes observed after IR. We added a reference in the introduction section showing the complexity of DNA lesions induced by IR (L129): Cadet et al., Cancer Letter, 2012. To clarify this point, we also modify a sentence of the results section line 154 that could be interpreted as opposing UV and IR in the formation of ssDNA gaps and UV. We also modify our conclusion about comparison between UV- and IR-induced HR in *rad55*∆ mutant (line 174). Finally, our microscopy observation of IR- and UV-induced RPA foci formation shows that UV induces more foci per cells compared to IR, confirming the conclusion from Wong et al that UV-induced damage takes place outside DSB repair center.

3) The results here suggest that in the absence of Rad55-Rad57, unstable Rad51 filaments cannot prevent the loading of the TLS polymerases, but what would be the mechanism of loading in the presence of RAD51 for Polζ and Polη ?. In human cells, it has been evidenced the ability of Polθ in removing Rad51 and RPA, but there is no evidence for other TLS polymerases to perform this task.

To which point TLS can displace Rad51 filaments, even stabilized by Rad55-Rad57 requires further study. The balance between HR and TLS is probably finely tuned in a way not yet described. However, our study highlights that UV-induced damages supported by HR can be supported by TLS (*rad55*∆ mutant is not sensitive to UV exposure) while some UV-induced damages supported by TLS cannot be supported by HR (*rev3*∆ mutant is sensitive to UV exposure). This point was made clear and explained in the results section line 276 and in the discussion section, line 436.

4) Did the authors test other TLS DNA polymerases ? Why only Polζ and Polη?

There are only three nuclear TLS in yeast, Polζ and Polη and Rev1 which is used mostly for the recruitment of Polζ and Polη.

5) In human cells; mutations in BRCA1 or BRCA2 (BRCA) is synthetic lethal with poly(ADP-ribose) polymerase inhibitors (PARPi) and such synthetic lethality is thought to derive from DSB repair BRCA function. It was recently reported by the Sharon Cantor’s lab (Cancer Res 2021, Mol cell 2021) instead that toxicity derives from replication gaps which characterize BRCA-deficient cells, are diminished upon resistance and restored upon resensitization. Such gaps are determinant of PARPi toxicity changing the paradigm for synthetic lethal interactions. These observations could be related to the present work and might be discussed.

These references were added in the last paragraph of the discussion section.
